# The dual orexin receptor antagonist suvorexant in alcohol use disorder and comorbid insomnia: A case report

**DOI:** 10.1002/ccr3.8740

**Published:** 2024-05-01

**Authors:** Erin J. Campbell, Yvonne Bonomo, Lisa Collins, Amanda Norman, Helen O'Neill, Amanda Streitberg, Kate Galloway, Andrew Kyoong, Andrew Perkins, Adam Pastor, Andrew J. Lawrence

**Affiliations:** ^1^ Florey Institute of Neuroscience and Mental Health Parkville Victoria Australia; ^2^ Florey Department of Neuroscience and Mental Health The University of Melbourne Melbourne Victoria Australia; ^3^ Department of Addiction Medicine St Vincent's Hospital Melbourne Melbourne Victoria Australia; ^4^ Department of Medicine The University of Melbourne Melbourne Victoria Australia; ^5^ Department of Respiratory and Sleep Medicine St Vincent's Hospital Melbourne Melbourne Victoria Australia; ^6^ Department of Respiratory and Sleep Medicine The Royal Melbourne Hospital Melbourne Victoria Australia

**Keywords:** alcohol use disorder, insomnia, orexin, relapse, sleep, suvorexant

## Abstract

**Key Clinical Message:**

This case suggests using dual orexin receptor antagonists to treat alcohol use disorder and comorbid sleep disorders may be effective, commencing treatment in withdrawal and continuing it to prevent relapse.

**Abstract:**

Effective medications for the treatment of alcohol use disorder are limited. This is partially due to the heterogenous nature of the symptomatology associated with alcohol use disorder and the abundance of presenting comorbidities. One common, and often overlooked, symptom that occurs during withdrawal of alcohol use is sleep disruption. Here, we report a case study of a participant with comorbid alcohol use disorder and insomnia. This participant was treated with a dual orexin receptor antagonist, suvorexant (Belsomra®), currently approved to treat insomnia. We demonstrate improvements in alcohol cravings, physical and psychological health, and sleep outcomes with treatment. These data support abundant preclinical and emerging clinical data in this space. The findings from this case report highlight the potential for suvorexant to treat comorbid alcohol use disorder and insomnia with fully powered, randomized controlled trials moving forward.

## 1 INTRODUCTION

Substance use disorders, including alcohol use disorder, are notoriously difficult to treat. Effective pharmacotherapeutic treatments are needed to aid behavioral interventions and formulate successful, holistic treatment regimes. Existing medications for the treatment of alcohol use disorder are suboptimal and there have been no new FDA‐approved medications for treatment in more than 18 years.^1^, ^2^, ^3^, ^4^, ^5^ One key symptom of alcohol use disorder, often critical for preventing subsequent relapse to alcohol use, is insomnia, during both withdrawal and post withdrawal. Persistent disruption in sleep activity is observed in individuals with alcohol use disorder, even after years of abstinence.^6^, ^7^ Improving sleep disturbance therefore may be a key component in assisting withdrawal and reducing subsequent relapse to drinking.

In 2014, the US Food and Drug Administration (FDA) approved Belsomra® (suvorexant), a dual orexin receptor antagonist, for the treatment of insomnia. Suvorexant has shown efficacy in the treatment of insomnia since its approval.^8^ While sleep disruptions are a common symptom of alcohol use disorder and suvorexant has shown promise at reducing sleep disturbances, the literature surrounding the effectiveness of suvorexant to treat comorbid alcohol use disorder and insomnia remains limited. We recently reviewed this literature and introduced our clinical trial plan on the use of suvorexant to treat alcohol use disorder with comorbid insomnia.^9^, ^10^ More recently, a milestone clinical study using suvorexant has reported increased sleep duration and reduced self‐reported withdrawal in a group of inpatients with opioid use disorder.^11^ Here, we report a case of the beneficial effects of suvorexant in the treatment of alcohol use disorder and comorbid insomnia. This case was part of our Phase II, placebo‐controlled, double‐blind randomized trial which was impacted by the COVID pandemic from 2020 to 2022 (NCT03897062) and ceased before completion.

## 2 CASE PRESENTATION

The information collected for this case report was approved by the Human Research Ethics Committee of St Vincent's Hospital, Melbourne. Participant 020 is a 31‐year‐old male, currently unemployed, and living in a private residence with his spouse. Participant 020 was admitted to the Drug and Alcohol withdrawal unit at St. Vincent's Hospital, Melbourne, Australia in March 2022. On admission, Participant 020 consumed 16 standard drinks per day and had mildly abnormal liver function (Table 1). He reported his last consumption of alcohol was 1 day prior to admission and his breath alcohol was 0.0% on admission. He was discharged after 7 days to his private residence and consented to remain in the study as an outpatient.

**TABLE 1:** Clinical liver function characteristics from Baseline to Week 9 follow‐up for Participant 020.

Liver function	(Normal limits)	Baseline	Week 9
Albumin	(35–50 g/L)	41	45
GGT	(0–50 U/L)	87	15
ALP	(30–110 U/L)	89	97
ALT	(0–40 U/L)	43	15
Bilirubin total	(1–20 μmol/L)	13	8

Abbreviations: ALP, alkaline phosphatase; ALT, alanine aminotransferase; GGT, gamma‐glutamyl transferase.

## 3 METHODS

For treatment, Participant 020 received one 20 mg tablet of suvorexant each evening, at around 8 p.m., for 13 weeks after which he was lost to follow‐up. The participant and administering personnel were blinded to treatment condition. Other medications during treatment included levetiracetam for epilepsy (500 mg, twice/day), thiamine prophylaxis against Wernicke–Korsakoff syndrome (100 mg three times/day during admission for alcohol withdrawal, 100 mg daily thereafter), Panadol (1 g 6 h for 3 days during Week 9 for symptoms of flu), ibuprofen (200 mg twice daily for 3 days during Week 9 for symptoms of flu), and diazepam for alcohol withdrawal (10–20 mg if Alcohol Withdrawal Score ≥ 10; total diazepam over course of 7 day admission for withdrawal was 145 mg). Participant 020 had not tried pharmacotherapies for alcohol use disorder such as naltrexone or acamprosate. Participant 020 reported daily tobacco use at the time of admission and intermittent use of cannabis (last use was 3 months prior to admission) and methamphetamine (last use was 9 years prior to admission). Alcohol use, psychological and physical health status, quality of life, sleep disturbance, and vital signs were monitored every 4 weeks post discharge.

## 4 CONCLUSION AND RESULTS

### 4.1 Alcohol use outcomes

Participant 020 reported abstinence from alcohol, confirmed by breath alcohol readings of 0.0% at all follow‐up visits. Follow‐up visits assessed alcohol and other drug use, sleep assessment, and adverse effects from the suvorexant. No other alcohol pharmacotherapies were offered. He was provided with supportive counseling but not structured psychological input (e.g., SMART Recovery, Motivational Interviewing, Cognitive Behavioral Therapy, or Alcoholics Anonymous). Final liver function data collected at Week 9 follow‐up revealed an overall improvement since admission to the trial (See Table 1).

**FIGURE 1:**
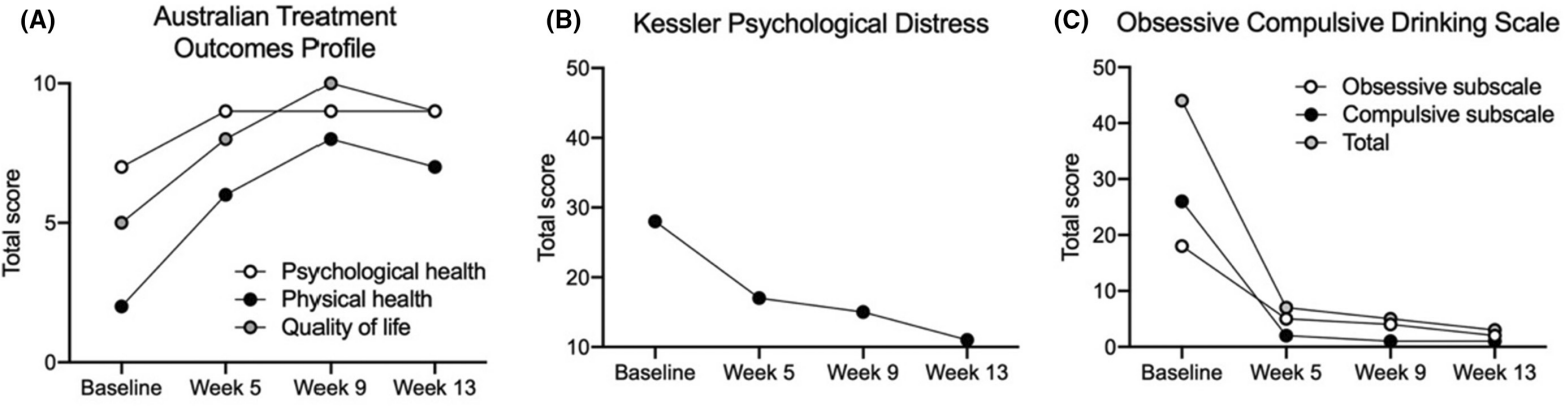
Self‐reported psychological distress, health, well‐being, and craving after suvorexant treatment in Participant 020 with comorbid alcohol use disorder and insomnia. Psychological and physical health status and quality of life increased over the treatment period (A). Psychological distress improved over the 13‐week treatment period (B). Self‐reported craving also reduced over the treatment period (C).

At baseline, Participant 020 reported poor physical health (2 on a scale of 0 [poor] to 10 [good]) and quality of life (5/10) on the Australian Treatment Outcomes Profile (ATOP) (Figure 1A).^12^ He reported moderate psychological health (7/10). At follow‐up sessions, he reported improved health and well‐being as scored on the ATOP (Figure 1A). Participant 020's Kessler psychological distress score (K10) was moderate on admission (28 out of total 50)^13^ but improved over the suvorexant treatment period (Week 5: 17/50; Week 9: 15/50; and Week 13: 11/50) (Figure 1B). Self‐reported craving was high on the Obsessive Compulsive Drinking Scale (44 out of total 56)^14^ on admission but reduced with treatment (Week 5: 7/56; Week 9: 5/56; and Week 13: 3/56) (Figure 1C).

### 4.2 Sleep disturbance outcomes

Participant 020 experienced an overall improvement in sleep outcomes following suvorexant treatment. Upon admission the Insomnia Severity Index indicated that Participant 020 had severe clinical insomnia (23 out of 28).^15^ Daytime sleepiness as assessed by the Epworth Sleepiness Scale was mild (12 out of 24).^16^ The Pittsburgh Sleep Quality Index also demonstrated poor sleep quality on admission by Participant 020 (17 out of 21).^17^

By Week 13 follow‐up, Participant 020 presented with no clinically significant insomnia based on the Insomnia Severity Index (2 out of 28). Daytime sleepiness was reported to be in the normal range (2 out of 24 with normal ranging from 0 to 10)^16^ at Week 13 follow‐up and sleep quality was reported to have improved substantially (6 out of 21).

### 4.3 Vital signs

Vital signs did not change over the course of suvorexant treatment (Table 2).

**TABLE 2:** Vital signs during the suvorexant treatment period.

Vital sign	Baseline	Week 5	Week 9	Week 13
Temperature (°C)	36.7	36.6	36.2	36
Pulse (beats/min)	90	99	75	102^a^
Respiratory (breaths/min)	16	16	16	16
Blood pressure: systolic	133	119	115	146
Blood pressure: diastolic	98	84	80	84

^a^ Participant 020 reports he walked fast to the Week 13 appointment.

Here, we present a case of an individual with alcohol use disorder and clinically significant comorbid insomnia. Participant 020 responded positively following 13 weeks of double‐blinded suvorexant treatment. This included complete abstinence over the treatment period and improvements in self‐reported physical and psychological health, as well as alcohol cravings. Sleep measures also improved during this period, with improved sleep quality, reduced daytime sleepiness, and reduced severity of insomnia.

Abundant preclinical research has identified a role for the orexin system in alcohol use disorder and related comorbidities.^18^, ^19^, ^20^, ^21^, ^22^, ^23^, ^24^ Thus, it is encouraging to observe the link between preclinical research and the initiation of human clinical trials in this area (NCT03897062, NCT04229095, and NCT05458609). While the case study described above does not allow for treatment recommendations, it does suggest potential for suvorexant in the treatment of comorbid alcohol use disorder and insomnia. Given the cost of alcohol misuse globally, examining the use of suvorexant to treat comorbid alcohol use disorder and insomnia through a fully powered phase II trial is warranted.

## 5 DISCUSSION

Insomnia features strongly in both withdrawal and relapse to alcohol consumption. In this case study, suvorexant was therefore administered during the withdrawal period and continued post withdrawal. Overall, this case suggests using dual orexin receptor antagonists to treat alcohol use disorder and comorbid sleep disorders may be effective, commencing treatment in withdrawal and continuing it to prevent relapse. Future work with a fully powered and controlled experimental design are needed.

## AUTHOR CONTRIBUTIONS

**Erin Campbell:** Formal analysis; writing – original draft; writing – review and editing. **Yvonne Bonomo:** Project administration; writing – review and editing. **Lisa Collins:** Data curation; writing – review and editing. **Amanda Norman:** Methodology; project administration; writing – review and editing. **Helen O'Neill:** Data curation; writing – review and editing. **Amanda Streitberg:** Investigation; project administration; writing – review and editing. **Kate Galloway:** Data curation. **Andrew Kyoong:** Methodology; writing – review and editing. **Andrew Perkins:** Formal analysis; writing – review and editing. **Andrew Lawrence:** Conceptualization; funding acquisition; investigation; project administration; writing – original draft.

## FUNDING INFORMATION

This work was supported by a Perpetual IMPACT philanthropic grant to AJL and EJC via the Percy Baxter Charitable Trust and a Victoria Medical Research Acceleration Fund 2 grant from the Victorian State Government to AJL and YB. We acknowledge the Victorian State Government Operational Infrastructure Scheme.

## CONFLICT OF INTEREST STATEMENT

All authors report no conflict of interest.

## CONSENT

*Ethical approval and consent to participate*: Approved by the Human Research Ethics Committee of St Vincent's Hospital, Melbourne. Project ID number 49730; HREC Ref: 271/18. The ability to provide informed consent was an inclusion criterion. *Consent for publication*: Written informed consent was obtained from the patient for publication of this case report and any accompanying images. A copy of the written consent is available for review by the publisher of this journal. Written informed consent was obtained from the patient to publish this report in accordance with the journal's patient consent policy.

## Data Availability

The data that support the findings of this study are available from the corresponding author upon reasonable request.
