# Correction to “The dual orexin receptor antagonist suvorexant in alcohol use disorder and comorbid insomnia: A case report”

**DOI:** 10.1002/ccr3.8961

**Published:** 2024-05-23

Campbell EJ, Bonomo Y, Collins L, et al. The dual orexin receptor antagonist suvorexant in alcohol use disorder and comorbid insomnia: A case report. Clin Case Rep. 2024; 12:e8740. doi:10.1002/ccr3.8740

In the article entitled “The dual orexin receptor antagonist suvorexant in alcohol use disorder and comorbid insomnia: A case report” that was previously published in Volume 12 Issue 5 of Clinical Case Reports, the address in the correspondence section for Andrew J. Lawrence has been corrected.

The correct address for Andrew J. Lawrence in the correspondence section should be: Andrew J. Lawrence, The Florey Institute of Neuroscience and Mental Health, Melbourne Brain Centre, The University of Melbourne, Parkville, Vic 3052, Australia.

We apologize for this error.

